# The NW extension of the Nahavand Fault along the Main Recent Fault, west Iran

**DOI:** 10.1371/journal.pone.0326603

**Published:** 2025-06-23

**Authors:** Reza Alipoor, Ehsan Aghayani

**Affiliations:** Department of Geology, Faculty of Basic Science, Bu-Ali Sina University, Hamedan, Iran; National Centre of Excellence (NCE) in Geology University of Peshawar, PAKISTAN

## Abstract

The MRF (Main Recent Fault) is an active fault zone in the western Iranian Plateau. The Nahavand Fault, an active 80-km segment of the MRF, lies between the Sahneh Fault to the northwest and the Qaleh-Hatam and Dorud faults to the southeast. The aim of this study is to examine the northwestern extension of the Nahavand Fault beneath Quaternary alluvial deposits. For this purpose, detailed field studies were conducted on a trench excavated perpendicular to the fault’s strike to determine the fault plane’s mechanism and characteristics. Additionally, data from the interpretation of two geoelectrical profiles were used to locate the surface trace of the fault beneath the alluvial deposits. The results indicate that, at the southeastern termination of the Nahavand Fault, the transpression stress regime has been overprinted by a transtensional regime. At its northwestern termination, the Nahavand Fault connects with the Sahneh Fault via a fault-bend pattern. The characteristics of the Nahavand Fault beneath the alluvial deposits of the Nahavand plain are N70°W/75°NE, with a rake of 15°. The Nahavand Fault extends northwestward with an updated length of approximately 80 km.

## Introduction

**Fig 1:**
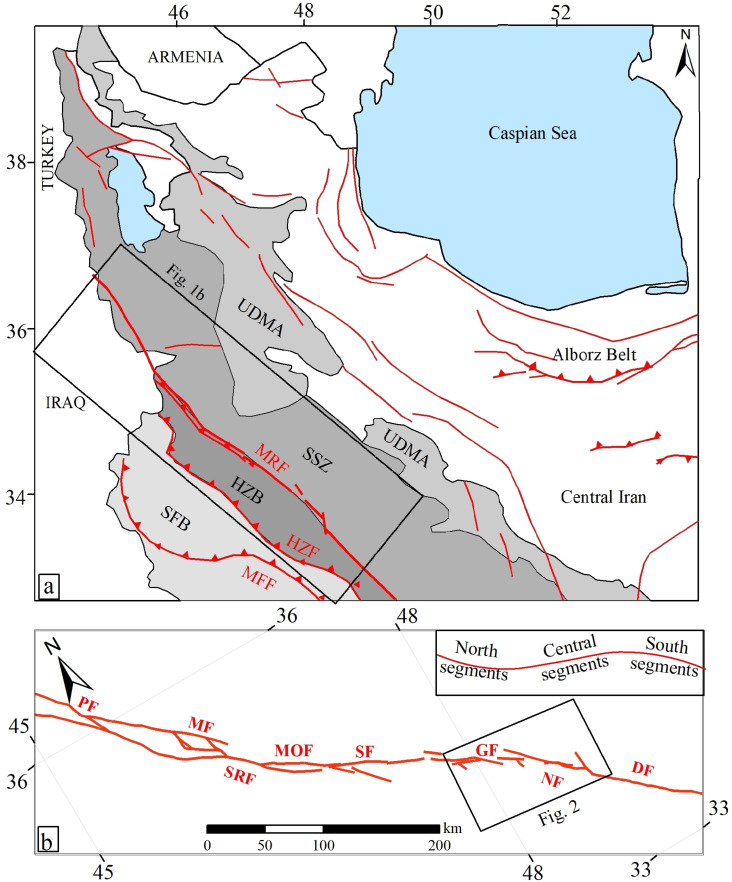
(a) Tectonic zoning map of the NW Iranian Plateau displaying major faults, with the location of the MRF highlighted, adapted and revised by the authors based on maps from the Geological Survey of Iran, used with permission citing the source [23,24]. The black rectangle indicates the location shown in Fig 1b. (b) The map of various segments of the MRF fault zone [1,25]. PF (Piranshahr Fault), MF (Marivan Fault), SRF (Sarvabad Fault), MOF (Morvarid Fault), SF (Sahneh Fault), GF (Garun Fault), NF (Nahavand Fault), DF (Dorud Fault). The location of the study area is indicated by a black rectangle.

The Main Recent Fault (MRF) is a key fault along the Zagros suture zone, separating the Eurasian and Arabian plates [1–7] (Fig 1a). This fault, with a northwest-southeast trend, consists of several connected fault segments [1,3,8–13] (Fig 1b). Geological and geomorphologic evidences, cosmogenic dating and Global Navigation Satellite System (GNSS) date indicate different slip rate along the MRF [3,4,7,14–21]. The MRF trends approximately 330° along the NW part (Marivan region), about 300° along the middle part (Sahneh region), and around 315° along the SE section (Dorud region) [3,22,23].

Several major earthquakes have occurred along or near the MRF, with seismic activity being more intense in the southeastern section than in the northwestern section of the fault [3]. Earthquake Focal mechanisms along the MRF suggest predominantly right-lateral strike-slip movement. The epicenters of earthquakes indicate that the most of seismic events near the MRF occur at depths of less than 15 km. Some of the earthquake focal mechanisms show normal faulting components, and the surface trace of the fault at the mountain-plain boundary creates scarps resembling the typical morphology of normal faults. Therefore, the normal fault component has contributed to the formation of certain linear plains, such as the Dorud and Nahavand plains.

The Nahavand fault, approximately 55 km in length, is one of the southern segments of the MRF. It extends from the southeast near the village of Vennai, west of Borujerd, and continues in an N320° direction to the village of Gusheh, northwest of Nahavand. [1]. Numerous studies have investigated the southeastern section of the Nahavand Fault near the Dorud Fault, whereas the northwestern termination of the Nahavand Fault and its connection to the Sahneh Fault remain poorly defined. In previous studies, the connection between the Nahavand and Sahneh fault segments has been mapped and interpreted as the en echelon pattern. In this research, using detailed geological filed studies and the geoelectric method, the connection between the Nahavand and Sahneh faults and the geometry and kinematics of the fault termination and its relationship with the Sahneh fault have been determined.

## Geological setting

The Zagros orogeny is the result of the convergence between the Arabian and Central Iranian plates from the Mesozoic to the present [2,26,27]. During this time, the Neotethyan Ocean closed, leading to the collision of the two continents [28]. This convergence has been oblique, leading to the development of numerous structural complexity [6,14,29]. The Zagros orogeny in west of Iran is divided into three belts: the Zagros Fold and Thrust Belt, the Zagros Suture Zone, and the Sanandaj-Sirjan Zone [30]. The suture zone consists of radiolarite, ophiolite, and Biston limestone. The Zagros Fold and Thrust Belt is further subdivided into the inner high Zagros belt and the outer simple Zagros belt. The High Zagros belt is a region characterized by elevated topography, comprising a thrust fault system dipping towards the northeast. This system has caused the overthrusting of deep Paleozoic rocks and shallower Mesozoic rocks onto Cenozoic strata [2,30]. The general trend of this belt is NW-SE, with a width ranging from 10 to 65 km, and it is situated along the SW boundary of the Sanandaj-Sirjan Zone. The northern boundary of the High Zagros High belt is marked by the Main Zagros Reverse Fault (MZRF), while its southern boundary is defined by the High Zagros Fault (HZF) [28,31]. Also, the oblique convergence of the Arabian and Eurasian plates is accommodated by a shortening component perpendicular to the mountain belt’s trend and a strike-slip component parallel to the trend, occurring along separate parallel faults [2,3]. The onset of oblique convergence between the Arabian and Eurasian plates has been considered to range from the Pliocene (3–5 million years ago) [3], to the early to middle Miocene (15–19 million years ago) [32], or even earlier [33,34], possibly as far back as the Late Cretaceous [10].

The shortening perpendicular to the belt’s trend has been accommodated by several processes, including the formation of reverse faults parallel to the MRF, folding and lithospheric-scale thickening, as well as the underthrusting of the Arabian lithosphere beneath the Central Iran plate [35,36]. It appears that most of the strike-slip movement is concentrated on the various segments of the MRF [1]. However, in some studies, the strike-slip component parallel to the orogen in the Zagros belt is not limited solely to the MRF fault zone and several external faults in the belt, such as the Marekhil and Ravansar faults, have also accommodated this oblique convergence [10]. Nevertheless, the MRF is one of the most seismically active fault systems in the northwestern part of the Zagros Mountains in western Iran, accommodating a significant portion of the right-lateral strike-slip motion associated with oblique convergence.

## Materials and methods

In this study, well-established methods in structural geology and morphotectonics, including detailed fieldwork at the surface and in trenches, and geological maps, were employed to examine the geometry, kinematics, and surface trace of fault systems, particularly in Quaternary deposits. Data related to the kinematics of the fault plane and fault slip, as well as the truncation of Quaternary deposits by faults, were gathered during field observations along the fault plane outcrops. Fieldwork for this study did not require formal permits, as all observations and photographs were conducted in publicly accessible locations with no legal or regulatory restrictions on entry or data collection. These sites, comprising open public lands, did not involve protected areas, private properties, or activities necessitating approval from governmental or institutional authorities. Additionally, electrical resistivity methods were employed to trace the fault’s effects beneath Quaternary deposits. This technique involves transmitting electric current to subsurface layers, creating a potential difference between two points, and estimating the resistivity of various layers at different depths. In surface electrical resistivity methods, as the depth of investigation increases, the vertical resolution decreases. To achieve higher accuracy and more detailed information, the spacing between electrodes should be reduced. Generally, the depth of study is dependent on the total distance of the electrode arrangement. Thus, by designing two electrical resistivity tomography profiles and utilizing a dipole-dipole array, the resistivity differences between layers and the fault’s effects were identified. Geoelectrical surveys conducted in this study required no permits, as all measurements were performed in publicly accessible areas with no regulatory restrictions on such activities.

## Results

The frequent occurrence of earthquakes, along with the displacement of Quaternary alluvial deposits, indicate evidence of the recent activity of the Nahavand Fault. The 1958 earthquake (Ms = 6.6) generated approximately 20 km of surface rupture along the southwestern side of the Nahavand-Firouzabad depression, indicating uplift along the southwestern segment of the Nahavand Fault [37]. The 1987 and 1998 earthquakes, both with magnitudes of Mw = 4.9, do not precisely align with surface fault traces in the Nahavand fault zone. In 2005, several earthquakes occurred along the Dorud and Nahavand fault segments, one of which took place on May 3rd with a magnitude of Mb = 4.9, near the southern termination of the Nahavand Fault, close to the city of Borujerd [22].

**Fig 2:**
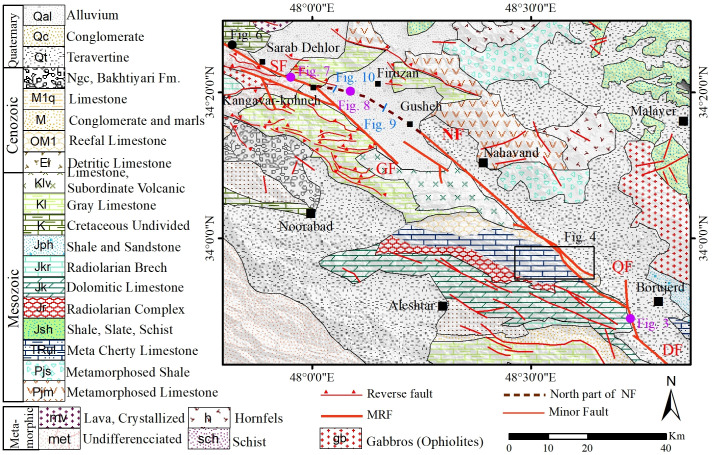
Geological map of the study area highlighting the Nahavand Fault, adapted and modified by the authors based on maps from the Geological Survey of Iran, used with permission citing the source [38,39]. The location of subsequent figures in the article are also indicated. The dashed line represents the NW extension of the Nahavand Fault, which is investigated in this study.

The Nahavand Fault is separated from the Dorud Fault at its southeastern termination by the Qaleh-Hatam Fault and connects with the Sahneh Fault at its northwestern termination (Fig 2). The Qaleh-Hatam Fault, trending nearly north-south, serves as a boundary separating the Nahavand Fault segment from the Dorud Fault segment. Movement along the Qaleh-Hatam Fault has displaced the southeastern section of the Nahavand Fault by approximately 3 km to the northeast relative to the Dorud Fault [1].

### The SE termination of the Nahavand Fault

**Fig 3:**
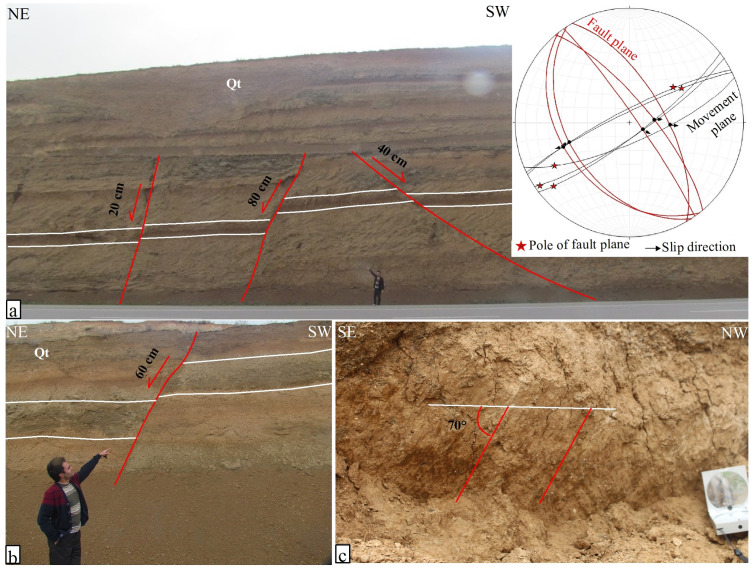
Field photographs and analysis of the Qaleh-Hatam fault zone, taken by the authors. (a) Normal faults within the Qaleh-Hatam fault zone, accompanied by a stereo-plot of the faults; the location is shown in Fig 2. (b) A normal fault with 60 cm of displacement within Quaternary strata. (c) A normal fault plane with slickenlines on the fault surface, indicating a normal faulting mechanism with a 70° slip rake angle.

At its southeastern termination, the Nahavand Fault connects to the Qaleh-Hatam Fault, which trends generally N-NW to S-SE. Along the Qaleh-Hatam fault zone, Quaternary conglomerates and horizontal clays have been displaced due to the fault’s activity. An analysis of drainage patterns and subsidence plains near the Dorud and Qaleh-Hatam faults likely indicates a normal faulting mechanism for the Qaleh-Hatam Fault [1]. Along the Nahavand to Borujerd main road, the activity of the Qaleh-Hatam fault zone has resulted in the formation of normal fault fracture zones within Quaternary deposits (Fig 3a). In this area, nearly horizontal clay layers of Quaternary age have been displaced by normal faults. The general trend of these normal faults is N-NW, ranging between N30°W and N40°W. The dip of these faults is either to the NE or SW. The first set of faults, which dip SW, are more gently dipping with angles between 40° and 45°. The second set of faults, dipping NE, are steeper, with dips between 65° and 85°. The displacement along these normal faults varies from 20 to 8 cm, with the steeper faults in the second set having greater displacements (Fig 3b). The slip rake angle, where measurable, is about 70° (Fig 3c).

**Fig 4:**
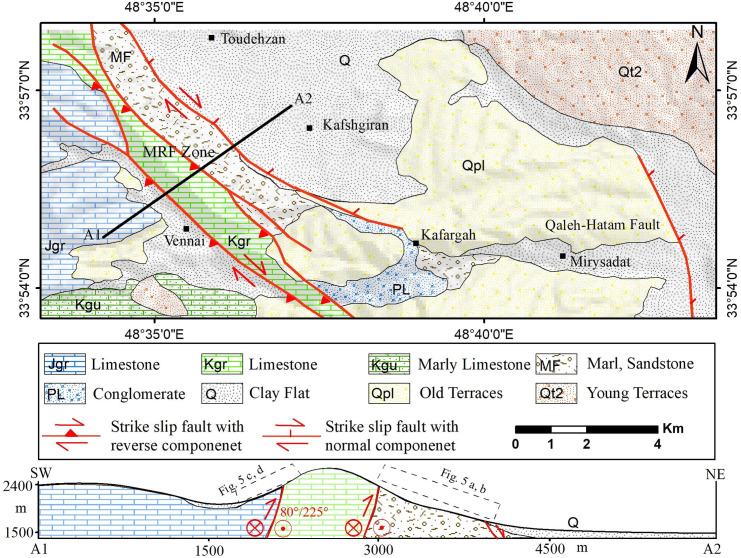
Geological map of the southeastern termination of the Nahavand Fault, adapted and modified by the authors based on maps from the Geological Survey of Iran, used with permission citing the source [39]. A geological cross-section with NE-SW direction, perpendicular to the Nahavand fault zone, indicates a transtensional and transpressional regime. The location of Fig 5 is indicated on the geological cross-section.

At the southeastern termination of the Nahavand Fault near Kafashgiran village, triangular facets or flat-iron landforms have developed along the fault zone. This area was extensively studied by [3], whose findings indicate that two parallel fault scarps have led to the formation of deep wine-glass-shaped valleys. These valleys suggest vertical and extensional slip components along this section of the Nahavand Fault. In this study, a more detailed examination of this part of the Nahavand fault zone has revealed additional evidence of deformation caused by fault activity. In addition to the northeast fault front in this area, where two sets of fault scarps have produced triangular facets, additional evidence of faulting has been observed in the rear and southwestern parts of this front. In this part of the Nahavand Fault, a fault zone with several closely spaced parallel fault segments has caused significant deformation of the region’s surface morphology. Field evidences in this area revealed not only a normal fault segment responsible for the formation of triangular facets but also two reverse oblique-slip fault segments that have thrust Cretaceous limestones over the Miocene marl and sandstone (Fig 4).

**Fig 5:**
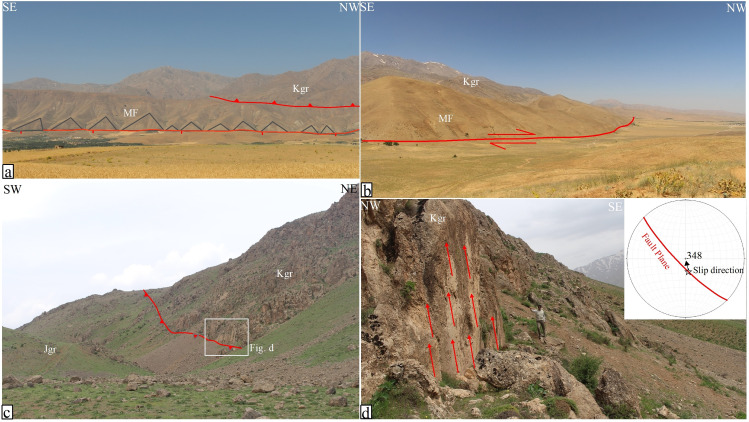
Field photographs of the Nahavand Fault zone, taken by the authors. (a) Triangular facets at the range front of the Nahavand Fault, indicating a normal fault component. (b) Right-lateral strike-slip movement along the fault at the boundary between the plain and Miocene limestones. (c) Thrusting of Jurassic limestones over Cretaceous limestones within the Nahavand Fault zone. (d) A reverse fault with characteristics of N45°W/80°SW and a 75° slip rake angle.

Therefore, the northeast fault segment, characterized by a normal slip component (Fig 5a) and right-lateral strike-slip motion along the boundary between the Miocene marl and sandstone units and the adjacent plain, appears to have contributed to recent deformation (Fig 5b). Additionally, the reverse oblique-slip fault, with attitude of N45°W/80°SW and a rake angle of 75° degrees, has resulted in the thrusting of the Jurassic limestones over the Cretaceous limestones (Fig 5c) and the thrusting of the Cretaceous limestones over the Miocene marl and sandstone (Fig 5d). Similar to the Piranshahr segment of the MRF [23], the southeastern termination of the Nahavand segment shows evidence of transtension superimposed on transpression. However, our field studies revealed no precise structural indicators of stress field changes, likely due to the dominance of erodible rocks obscuring fault plane evidence. Transpression is indicated by dextral strike-slip fault planes, while transtension is inferred from triangular facets, suggesting a potential stress shift linked to the Arabian-Eurasian collision dynamics.

### The NW termination of the Nahavand Fault

The main trend of the MRF shifts from southeast to northwest. At the southeastern termination near the Dorud fault segment, the general trend is about N45°W, while in the central section, around the Sahneh fault segment, it trends approximately N60°W. Farther northwest, near Marivan, the trend shifts to approximately N30°W [3]. The Sahneh Fault, exceeding 100 km in length and trending generally between N60°W and N56°W, links the Morvarid Fault segment in the northwest to the Nahavand and Garun fault segments in the southwest. The Sahneh fault is divided into three sections: Northwest, Central, and Southeast, each having nearly equal lengths. The southeastern segment of the Sahneh Fault begins at its intersection with the Garun Fault and extends along the Gamasiab Valley.

**Fig 6:**
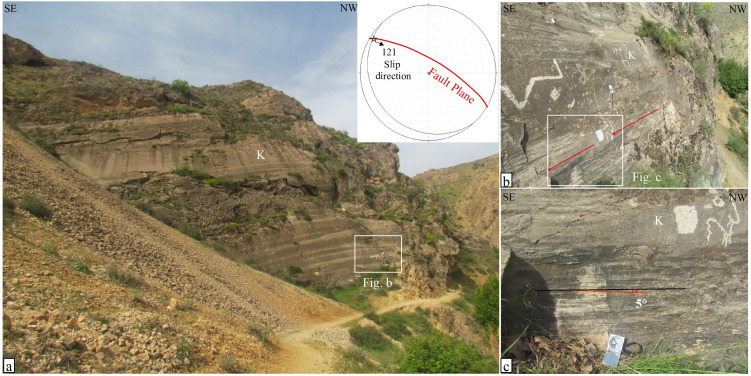
Field photographs of the Sahneh fault plane, taken by the authors. (a) The Sahneh fault plane in massive Cretaceous limestones, accompanied by a stereo-plot of the fault. (b) and (c) Close-up views of the Sahneh fault plane, showing right-lateral strike-slip movement with a 5° slip rake angle.

In this study, detailed field observations were conducted along the southwest segment of the Sahneh Fault to investigate the junction between the northwest termination of the Nahavand Fault and the Sahneh Fault. Since the southwest segment of the Sahneh Fault cuts through Cretaceous limestone sequences, detailed evidence of the fault plane’s geometry and characteristics can be accurately assessed. However, the northwest termination of the Nahavand Fault mostly traverses Quaternary deposits, complicating the identification of faulting evidence in the field. A well-exposed section of the Sahneh fault zone can be observed in its southeast segment, northwest of Sarab-Dehler village (Fig 2). This outcrop is one of the few exposures of the MRF fault zone in the western Iranian plateau, and it is introduced for the first time in this study (Fig 6a). In this part of the MRF fault zone, within Cretaceous limestone units, the Sahneh fault plane trends N60°W and dips 74° towards the northeast (Fig 6b). Additionally, the slip rake angle is nearly zero, approximately 5° to the north, indicating right-lateral strike-slip motion with a reverse component (Fig 6c).

**Fig 7:**
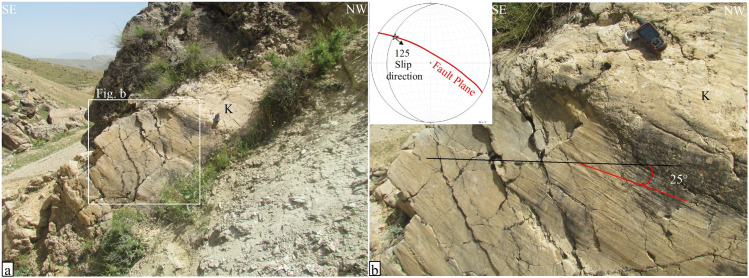
Field photographs of the Sahneh fault plane, taken by the authors. (a) Close-up view of the Sahneh fault plane in Cretaceous limestones. (b) The fault plane with a stereo-plot showing fault characteristics of N60°W/78°NE and a 25° slip rake angle; the location is indicated in Fig 2.

Additionally, in the southeast part of Sarab-Dehler village, near the southeast termination of the Sahneh Fault, another exposure of the fault plane within Cretaceous limestone reveals recent deformation (Fig 2). In this section of the fault, near its southeast termination, a slight change in both the fault dip and the slip rake angle is observed (Fig 7a). The fault plane’s orientation remains similar to the previous exposure, trending N60°W, but the fault dip has increased to 78° towards the northwest (Fig 7b). The slip rake angle, which was nearly horizontal and close to zero in the previous exposure, has increased to 25° toward the north. Therefore, in the southeast segment of the Sahneh Fault, both the fault dip and the slip rake angle increase progressively from northwest to the southeast termination of the fault.

### The junction of the Sahneh and Nahavand faults

**Fig 8:**
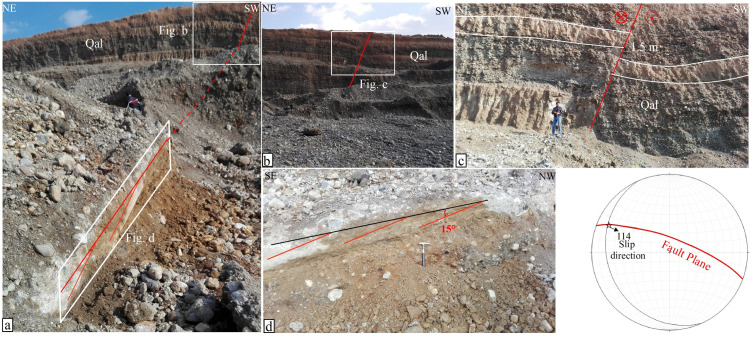
Field photographs of the Nahavand Fault, taken by the authors. (a) A trench excavated perpendicular to the Nahavand Fault, showing the fault plane in Quaternary alluvium. (b) Displacement of Quaternary marls and conglomerates by the Nahavand Fault. (c) Approximately 1.5 m separation in Quaternary alluvium caused by right-lateral strike-slip motion of the Nahavand Fault. (d) Stereo-plot and slip rake angle of the fault, approximately 15°, indicating a right-lateral strike-slip mechanism with a reverse component.

The most comprehensive study on the mapping of different segments of the MRF fault zone was conducted by [1]. In this research, detailed maps of the various segments of the MRF fault, between latitudes 33° and 35° N, were constructed. These maps have since served as a base map for subsequent studies, where various researchers have investigated the geometry, kinematics, and seismicity of the MRF fault zone (e.g., [3,7,22]. In these maps, the northwest termination of the Nahavand Fault is tentatively represented by a dashed line extending toward Gusheh village, while the area between the Nahavand Fault and the Sahneh Fault is depicted with an en-echelon pattern. This pattern, along with the surface trace of the Nahavand and Sahneh faults, is shown in Fig 2. The northwest termination of the Nahavand Fault, from near Gusheh village to the Sahneh Fault, is mostly covered by Quaternary deposits and agricultural plains. This makes it hard to study fault geometry with traditional field methods. Therefore, in this study, a large trench, measuring approximately 15 meters in depth, 50 meters in length, and 60 meters in width, was excavated along the fault zone to investigate the geometry of the Nahavand Fault beneath the alluvial cover. (Fig 8a). In the trench walls, Pleistocene marls and conglomerates are exposed, with the marls appearing in light cream color and the conglomerates ranging from light to dark brown (Fig 8b). Generally, considering the displacement of the Pleistocene marl and conglomerate units, the fault separation is observed as a reverse fault with approximately 1.5 m of reverse displacement (Fig 8c). This separation, which has caused the uplift of the hanging wall on the northeast side of the fault, can be readily measured in the light cream colored marl unit. The rake angle of the slip vector is approximately 15°, indicating a strike-slip fault where the right-lateral movement with a reverse component has caused the apparent separation of the fault plane in the trench wall to resemble a reverse fault (Fig 8d).

However, upon detailed examination of the fault plane and measuring the slip rake angle, the mechanism of the Nahavand fault in this trench is identified as a right-lateral strike-slip fault with a reverse component. In this significant exposure of the Nahavand fault plane, the fault plane’s trend is N70°W with a dip of 75° toward the northeast. Considering that this significant exposure of the Nahavand fault is located in the transitional area between the Sahneh and Nahavand faults, this study utilized the geoelectric method to identify the surface trace of the Nahavand fault beneath the Quaternary alluvium. In fact, two geoelectric profiles with a northeast-southwest orientation have been designed, one located northwest and the other southeast of the fault exposure in the trench.

In recent years, geophysical methods have been increasingly used to identify subsurface structures, particularly active fault zones [40–43]. Among the common geophysical methods, the geoelectric method is widely applied, as it can detect faulted zones in both rock sequences and alluvial deposits by displaying these zones as resistivity anomalies. In fact, rock sequences on either side of the fault exhibit similar electrical resistivity, while the faulted and fractured zones reveal anomalies in resistivity.

The potential limitations of geophysical resistivity methods for detecting faults beneath Quaternary deposits stem from several interconnected factors. The inherent heterogeneity of these deposits, such as alluvial soils or surface layers with varying grain sizes and moisture content, can scatter or attenuate electrical signals, leading to reduced resolution in identifying faults [44]. Thick Quaternary layers further complicate matters by limiting signal penetration, making it difficult to detect deeper faults as the electrical current struggles to reach sufficient depths [45]. Environmental conditions, like high moisture or salinity within the deposits, alter electrical resistivity, which can obscure the data and make interpretation challenging, as these variations might be mistaken for geological features rather than surface effects. The complex texture of Quaternary sediments also contributes to signal dispersion, lowering the clarity of subsurface imaging and hindering the ability to distinguish fault boundaries, which rely on contrasts in resistivity. Since resistivity methods are generally more effective at shallow to moderate depths, their performance diminishes under thick overburden, potentially missing deeper structures entirely [45].

The electrical resistivity method is based on the distribution of electrical potential within subsurface sequences through current-carrying electrodes, and it depends on the resistivity of rock sequences and alluvial deposits [46,47]. In this method, by transmitting electrical current into subsurface sequences and creating a potential difference between two points, electrical resistivity is calculated at various depths along the profile.

The geoelectrical method, such as electrical resistivity, is widely used in geophysics to study subsurface properties, but it has limitations. Depth resolution decreases as signals penetrate deeper, scattering and reducing the ability to distinguish small or subtle changes in resistivity, with effectiveness tied to electrode configurations. Noise from environmental sources, like power lines or industrial activity, and poor electrode contact in dry or rocky terrains can distort data, making interpretation difficult [48]. Interpretation errors arise because resistivity data can be non-unique, meaning different subsurface models might produce similar outcomes, compounded by variables like soil moisture or temperature changes and overly simplistic assumptions about uniform layering that further reduce accuracy [49]. Combining this method with others, such as seismic surveys, and careful experiment design can help, but success depends on environmental conditions and the interpreter’s skill. In this study, geoelectrical surveys were conducted in a relatively quiet environment, far from power lines, industrial equipment, vehicle traffic, and similar disturbances, resulting in a very low noise level. Potential errors were also minimized by calibrating the equipment. Furthermore, the relatively horizontal layers of Quaternary deposits contributed to reducing errors.

The geoelectrical method, or electrical resistivity method, can be implemented using various electrode arrays. These arrays are named differently depending on the configuration of the current and potential electrodes on the ground surface, and the calculation of electrical resistivity varies accordingly. The method used in this study is the dipole-dipole array, which is widely applied due to its ability to resolve vertical structures and its low electromagnetic coupling between the current and potential loops. For this purpose, two profiles were collected using the dipole-dipole method, perpendicular to the fault trend. In fact, in this research, to reduce the limitations of the geoelectric method in the detection of faults under Quaternary deposits, dipole-dipole electrode arrays, adherence to noise reduction measures (as explained above), and precise interpretation have been employed.

Therefore, considering the outcrop of the Nahavand fault plane in the excavated trench (Fig 8), which has an approximate dip of 75°, the dipole-dipole method is suitable for detecting the trend of this fault. In this study, the GEOB model 2007A geoelectric device was utilized, equipped with a rechargeable battery providing two energy levels of 100 and 200 watts for data acquisition. This configuration allowed sufficient energy for subsurface explorations up to a maximum depth of 40 m. Two geoelectric profiles were selected to investigate the surface trace of the Nahavand fault, as shown in Fig 2. The first profile, located northeast of Gusheh village and south of Firuzan village, has a flat topography, requiring no topographic correction during data processing. This profile strikes N20ºE, with a length of 200 m and electrode spacings of 20 and 30 m. The acquired data were processed using the RES2DINV software, which automatically discretizes the subsurface into rectangular blocks and employs the least squares inversion method to calculate the electrical resistivity for each block. The inversion process was iterated until convergence, with absolute error values (Iteration 3 Abs error) of 5% and 6% achieved for the two profiles, respectively, indicating acceptable data fit and model reliability. Unlike traditional RMS error, the absolute error metric in RES2DINV reflects the difference between observed and calculated apparent resistivity values after three iterations, ensuring computational efficiency while maintaining accuracy. To assess model resolution, the block discretization and resistivity contrasts were evaluated, confirming that the model effectively resolves fault-related features down to the maximum depth of 40 m, though resolution naturally decreases with increasing depth due to the diffusion of electrical current. Depth accuracy was constrained by the electrode spacing (20–30 m) and energy input, ensuring reliable detection of subsurface structures within the upper 40 m, consistent with the geological context of the alluvial deposits and underlying fault.

**Fig 9:**
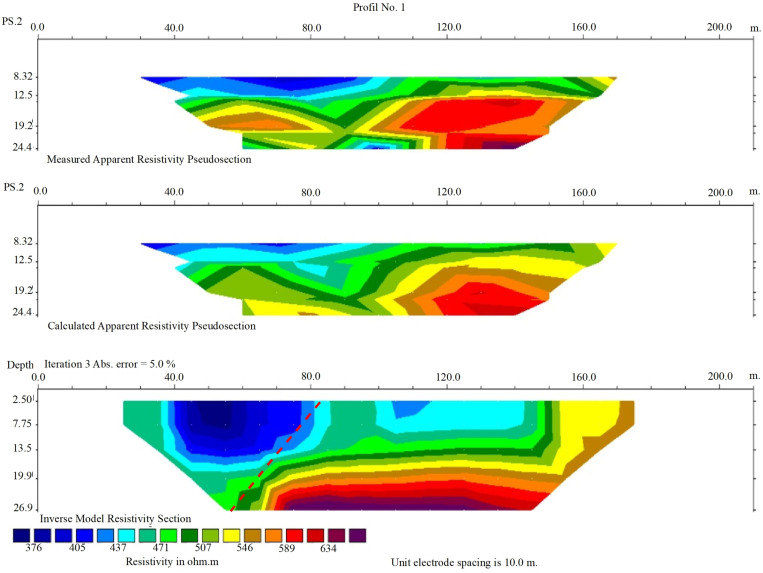
Geoelectric profile (Profile No. 1) perpendicular to the Nahavand Fault, showing subsurface resistivity over a 200-meter transect. Includes Measured, Calculated, and Inverse Model Resistivity Sections (iteration 3, error = 5.0%), with resistivity ranging from 376 to 634 ohm·m. Resistivity is lower on the left (hanging wall) and higher on the right (footwall) of the fault. Unit electrode spacing is 10.0 meters.

In first profile, a reduction in resistivity is observed at shallow depths, corresponding to the marly layers in the hanging wall of the fault (Fig 9). As illustrated in Fig 8c, a thick sequence of light-colored marl lies beneath the conglomerate and marly sequence in the fault’s hanging wall. Due to the salts present in these marls, they exhibit high electrical conductivity and, consequently, lower electrical resistivity. At greater depths, on the left and northeast sides of the profile, a significant variation in resistivity is observed, indicating that the sequences have been disrupted by the Nahavand Fault.

**Fig 10:**
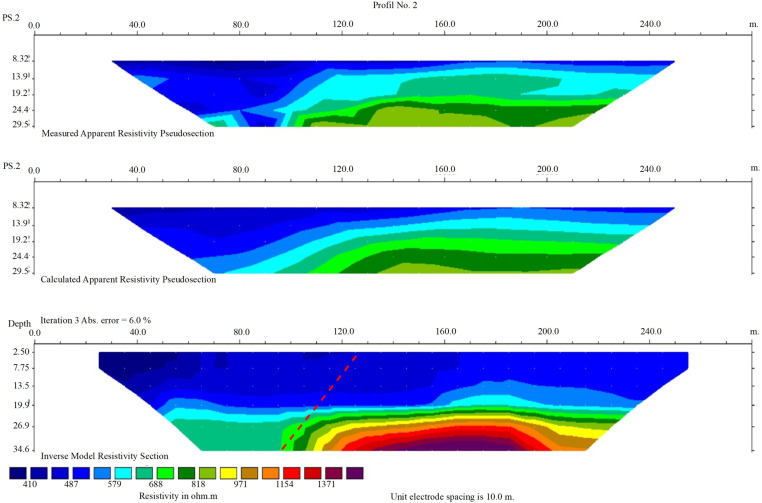
Second geoelectric profile (Profile No. 2) perpendicular to the Nahavand Fault, showing subsurface resistivity over a 240-meter transect. Includes Measured, Calculated, and Inverse Model Resistivity Sections (iteration 3, error = 6.0%), with resistivity ranging from 410 to 1371 ohm·m. The dashed lines indicate the location of the Nahavand Fault, with lower resistivity on the left (hanging wall) and higher on the right (footwall), reflecting conductive and resistive layers, respectively. Unit electrode spacing is 10.0 meters.

The second profile is located to the NW of the trench, where the Nahavand fault outcrop was observed. This profile was selected west of Firuzan village and southeast of Kangavar-Kohneh village. The length of this profile is 280 meters, allowing for data collection at greater depths. The electrode spacing was 20 meters, and 50 data points were recorded. The overall orientation of this profile was chosen as N15°E (Fig 10). The general view of this profile is similar to the first, with some variations in resistivity. In the fault’s hanging wall, on the northeast side, resistivity generally decreases. At depths greater than 25 meters, a noticeable change and decrease in resistivity can also be observed on the left side of the profile. However, at shallower depths near the surface, the resistivity reduction is not exactly similar to the first profile, which is likely due to variations in the thickness of the conglomerate sequence and marly interlayers near the surface.

## Discussion

In this study, to accurately examine the geometry and kinematics of the Nahavand Fault, both the southeast and northwest terminations of the fault were investigated using field evidence. The primary focus was to determine the connection area between the Nahavand Fault and the Sahneh Fault at the northwest termination of the Nahavand Fault. Previous studies had mapped various segments of the MRF, including the Dorud Fault, Nahavand Fault, and Sahneh Fault, but the northwest termination of the Nahavand Fault, near Gusheh village, had only been mapped tentatively.

**Fig 11:**
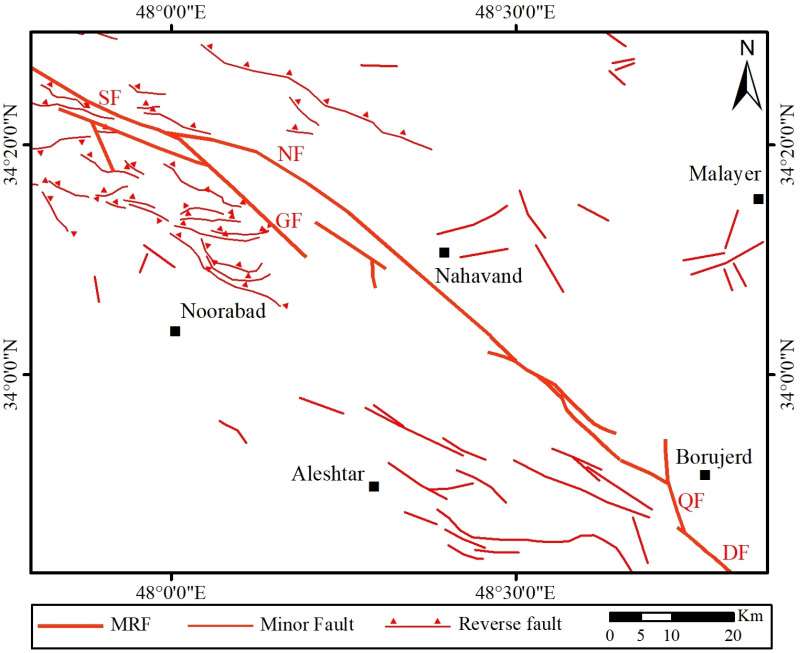
Fault map of the study area showing the newly identified surface trace of the Nahavand Fault, extending for 80 km, adapted and modified by the author from the Geological Survey of Iran fault map [38,39] used with permission citing the source, with modifications based on this study.

Initially, a trench was excavated in the area between the termination of the Nahavand Fault near Gusheh and the Sahneh Fault near the Kangavar-Kohneh to study the geometric and kinematic characteristics of the Nahavand Fault. This trench shows fault displacement in Quaternary deposits, indicating tectonic activity. As these deposits cover a wide timeframe (2.58 million years to present), the deformation could reflect either recent seismic activity or paleo-seismic events. Without precise dating, this remains unclear, and future geochronological studies (e.g., radiocarbon or luminescence dating) are needed to determine the timing. Based on the precise location of the fault beneath the alluvial deposits, two geoelectric profiles were selected in the southeast and northwest parts of the trench. In both profiles, resistivity anomalies indicated the influence of the fault on Quaternary deposits. Consequently, in the transitional zone between the Nahavand and Sahneh faults, the location of the Nahavand Fault beneath the alluvial layers was identified at three locations. By connecting these points and plotting the fault’s trace, the northwest termination of the Nahavand Fault was established from near Gusheh village to Kangavar-Kohneh village. Finally, the map of the surface trace of the active Nahavand Fault was revised, showing that instead of an en-echelon pattern, the fault is connected to the Sahneh Fault through a fault bend structure (Fig 11).

Additionally, the length of the Nahavand Fault, previously estimated at 55 km in earlier studies, extends approximately 25 kilometers beneath the Quaternary alluvial deposits, bringing the total length of the fault to around 80 km. Based on seismic evidence and field studies, it appears that the right-lateral strike-slip movement of the Nahavand Fault is accompanied by a normal slip component. However, at the northwest termination, near the fault bend, both the fault’s trend and slip component change, with the normal slip transitioning into a compressional component. This compressional feature is visible at the fault plane measured in the trench.

## Conclusion

The Nahavand active fault is one of the main segments of the MRF fault zone, located between the Sahneh Fault to the northwest and the Dorud and Qaleh-Hatam segments to the southeast. The Qaleh-Hatam fault zone, with a general NW-SE trend, has caused the formation of normal faults in Quaternary deposits. Additionally, evidence of normal components of the right-lateral strike-slip movement is visible through the formation of triangular facets, while compressional components are reflected in the formation of reverse faults. These indicate an overprinting of transpression by transtension. Field evidence suggests that the characteristics of the Sahneh fault segment to the northwest of the Nahavand fault are N60°W/74°NE with a 5° slip rake angle, indicative of right-lateral strike-slip movement with a reverse component. The northwestern termination of the Nahavand fault (close to the Gusheh village) to the SE termination of the Sahneh fault (close to the Kangavar-Kohneh village) is covered by Quaternary alluvium. Interpretation of two geoelectric profiles in this part of the MRF fault zone indicates that the Nahavand fault is connected to the Sahneh fault through a fault-bend pattern. Detailed field studies in a trench excavated perpendicular to the Nahavand fault reveal fault plane characteristics of N70°W/75°NE with a 15° slip rake angle, indicating right-lateral strike-slip movement with a reverse component. Where the Nahavand fault connects to the Sahneh fault, the right-lateral strike-slip movement with a normal component at the fault bend, overprinted by the right-lateral strike-slip movement with a compressional component. Therefore, based on the findings of this study, the revised length of the Nahavand Fault increases from 55 to 80 km, and it connects to the Sahneh fault segment.
